# The complete chloroplast genome sequence of *Lavandula dentata* (*Lamiaceae*) and its phylogenetic analysis

**DOI:** 10.1080/23802359.2019.1623107

**Published:** 2019-07-10

**Authors:** Hui Li, Jingrui Li, Hongtong Bai, Lei Shi, Huafang Wang

**Affiliations:** a College of Biological Sciences and Biotechnology, National Engineering Laboratory for Tree Breeding, Beijing Forestry University, Beijing, China;; b Key Laboratory of Plant Resources and Beijing Botanical Garden, Institute of Botany, Chinese Academy of Sciences, Beijing, China;; c University of Chinese Academy of Sciences, Beijing, China

**Keywords:** Chloroplast genome, *Lavandula dentata*, de novo sequencing, phylogenetic relationship

## Abstract

Fringed lavender (*Lavandula dentata* L., Lamiaceae) is a species of flowering plant commonly grown as an ornamental plant for its finely-toothed leaves, bulrush-like flower spikes, and light-woolly texture. Besides, *L. dentata* is distinguished by the high percentage of 1,8-cineole and β-pinene in its essential oil, endowing its highly commercial value. The complete chloroplast (cp) genome of *L. dentata* (GenBank accession number: MK791204) consists of 151,696 bp and includes a pair of inverted repeats (IR) of 25,608 bp separated by the large single copy region (LSC) and small single copy region (SSC) regions of 82,897 and 17,583 bp, respectively. The genome with an overall GC content of 37.9% hosts 114 unique genes including 80 protein-coding genes, 30 tRNA, and four rRNA (rrn5, rrn4.5, rrn16, and rrn23). Phylogenetic analysis of the *Lamiaceae* based on 16 plastome sequence data gives a hint at the basic structure of the *Lamiaceae*.

*Lavandula dentata*, also known as fringed lavender is a species of flowering plant in the *Lamiaceae* family and grows wild along the Mediterranean coast of Spain at 50–100 m. It is commonly grown as an ornamental plant for its finely-toothed leaves, bulrush-like flower spikes, and light-woolly texture. Besides, *L. dentata* is distinguished by the high percentage of 1,8-cineole and β-pinene in its essential oil, endowing its highly commercial value. It has also been used in folk medicinal practices for its anti-spasmolytic properties with coumarin and some triterpenoid constituents (Khalil et al. 1979).

Plants of *L. dentata* were introduced from the Czech Republic and cultivated at nursery of Beijing Botanical Garden (N39°59′16.83″ E116°12′34.98″). The specimen was kept at the Chinese national herbarium, Institute of Botany, Chinese academy of sciences with accession number 02328594. Total DNA was isolated from fresh leaves following the manufacturer’s protocol of DNA extraction kit (TSINGKE). Whole genome sequencing using an Illumina Hiseq 2000 PE150 platform. Approximately 5 Gb clean data were obtained and de novo assembled using NoVoPlasty 2.7.1 (Dierckxsens et al. 2017).

The plastome of *L. dentata* with 37.9% GC content is 151,696 bp in length and highly conserved in structure, sharing the common typical quadripartite structure comprising two copies of IR (25,608 bp) separated by the LSC (82,897 bp) and SSC (17,583 bp) regions (GenBank accession number: MK791204). A total of 114 unique genes were annotated, including 80 protein-coding genes (PRGs), 30 tRNA, and four rRNA (rrn5, rrn4.5, rrn16, and rrn23). In the two IR regions, 18 genes were duplicated including six PRGs, eight tRNAs, and four rRNAs. Seventeen genes contained introns, 14 (eight protein-coding and six tRNA genes) of which contained one intron and three of which (*rps12*, *ycf3*, and *clpP*) contained two introns. The *rps12* gene is a trans-spliced gene, three exons of which were located in the LSC region and IR regions, respectively. The complete gene of matK was located within the intron of trnK-UUU. Two pseudogenes, *ycf1* and *rps19*, were identified, located in the boundary regions between IRB/SSC or IRA/LSC.

To validate the phylogenetic position of *L*. *dentata*, a maximum-likelihood (ML) phylogenetic tree was reconstructed with RAxML (Stamatakis et al. 2008) using other 16 complete chloroplast genomes of *Lamiaceae* rooted by *Lindenbergia philippensis* and *Paulownia tomentosa* from the family *Paulowniaceae* (Figure 1). The phylogenetic analysis showed that all representatives of *Lamiaceae* were clustered into two clades with high bootstrap support values. One comprising the orders *Ocimeae* and *Mentheae*, *L. angustifolia* and *L. dentata* were clustered together first both belong to subfamily *Lavanduloideae*, then clustered with *Ocimum basilicum*, which belongs to subfamily Ocimoirleae. These three species both belongs to *Ocimeae.* While *Salvia bulleyana*, *Salvia przewalskii*, *Salvia miltiorrhiza*, *Salvia japonica*, *Rosmarinus officinalis*, and *Mentha spicata*, are members of the *Mentheae. Ajuga reptans*, *Premna microphylla*, *Scutellaria baicalensis*, *Scutellaria lateriflora*, *Leonurus japonicus*, *Galeopsis tetrahit*, *Lamium galeobdolon*, and *Lamium album* were clustered for the second clades.

**Figure 1.**
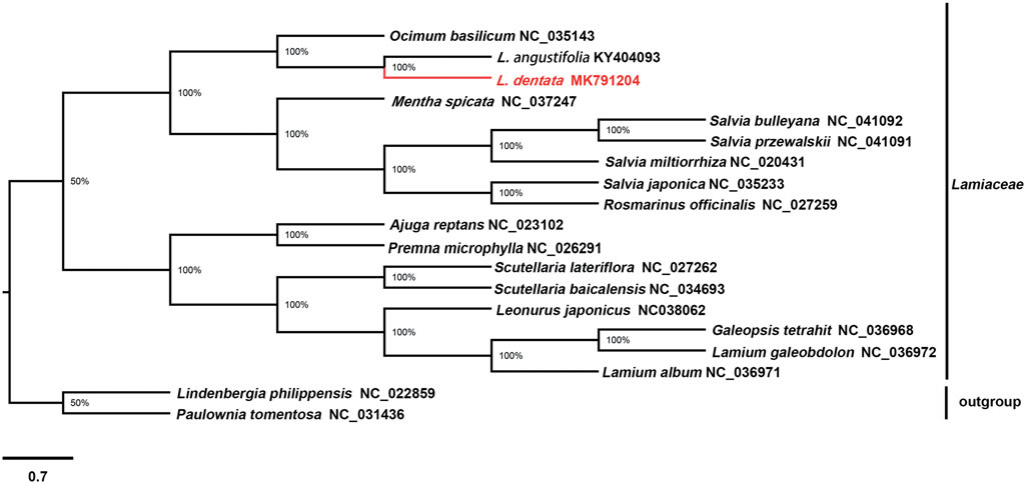
Molecular phylogeny of *Lavandula dentata* and other 16 related species in *Lamiaceae* rooted by *Lindenbergia philippensis* and *Paulownia tomentosa* from the family *Paulowniaceae* based on plastome sequence data. The complete chloroplast genome is downloaded from NCBI database and the maximum-likelihood (ML) phylogenetic tree is constructed by RAxML software. *Lavandula dentata* is labelled with red bold in the study. Bootstrap values are shown as percentages.

## References

[CIT0001] Dierckxsens N, Mardulyn P, Smits G 2017 NOVOPlasty: de novo assembly of organelle genomes from whole genome data[J]. Nucleic Acids Res. 45:e18. 28204566 10.1093/nar/gkw955 PMC5389512

[CIT0002] Khalil AM, Ashy MA, El-Tawil BAH, Tawfiq NI 1979 Constituents of local plants: 5. The coumarin and triterpenoid constituents of *Lavandula dentata* L. plant. Pharmazie. 34:564–565.

[CIT0003] Stamatakis A, Hoover P, Rougemont J 2008 A rapid bootstrap algorithm for the RAxML web servers. Syst Biol. 57:758–771. 18853362 10.1080/10635150802429642

